# Research on Embedded Multifunctional Data Mining Technology Based on Granular Computing

**DOI:** 10.1155/2022/4825079

**Published:** 2022-06-20

**Authors:** Juan Li, Xianghong Tian

**Affiliations:** ^1^School of Computer Engineering, Jinling Institute of Technology, Nanjing, Jiangsu 211169, China; ^2^Jiangsu Provincial Key Laboratory of Data Science and Intelligent Software, Nanjing, Jiangsu 211169, China

## Abstract

Due to the influence and limitations of the multisourced, heterogeneous, and unbalanced characteristics of embedded multifunctional data, the application effect of the current data mining technology is not good, and the accuracy is low. To solve the above problems, an embedded multifunctional data mining technology based on granular computing was studied. According to the three characteristics of embedded multifunctional data, preprocessing such as data reduction, data standardization, and data balance were implemented. We implemented data granulation for the preprocessed data and calculated the data granulation characteristics, including offset, particle density, and intraparticle interval. Taking granular features as the input content, embedded multifunctional data mining was realized by using a neural network to complete the objectives of data classification, anomaly detection, fault identification, and so on. The experimental results showed that the anomaly mining results of each type of data mining were greater than 0.9, indicating that the accuracy of the mining technology is high.

## 1. Introduction

Data mining refers to mining hidden rules or features from massive data for decision analysis. For example, in fault identification, data mining can judge the collected data and perform user classification to help develop sales strategies [1]. Therefore, data mining is an important aspect of big data processing and is the focus of research in the current information networks. To date, although big data research has achieved great success and significant achievements have been made in big data mining [2], there are still some problems that need to be deeply studied, among which embedded multifunctional data mining is a difficult point. Embedded multifunctional data are a kind of data with multiple description functions stored in a number of heterogeneous sources. The typical characteristics of such data are that it is multisourced, heterogeneous, and imbalanced. The existence of these three features makes data mining face great difficulties, and its accuracy and efficiency are greatly limited. Due to the influence and limitations of the multisourced, heterogeneous, and unbalanced characteristics of embedded multifunctional data, the current data mining technology has low accuracy and poor application effects in practical applications. Based on the above background, how to improve the accuracy and efficiency of embedded multifunctional data mining has become the focus of the current research.

At present, there are many studies on data mining techniques. For example, Wang Zhanping et al. [3] applied data mining technology to container shipping price prediction. In their research, based on the collected historical container shipping price time-series data, the container shipping price prediction model was established based on the GBDT algorithm to realize the data mining. Zhang Lili et al. [4] applied data mining technology to aviation customer classification. In the research, based on the passenger flight records of airlines, customer loss was predicted using the decision tree method, and the K-means clustering algorithm was used to classify customer categories and explore customer value, which provides a reliable basis for formulating effective marketing strategies and improving the economic benefits of airlines. Ma Lili et al. [5] applied a data mining technology map to abnormal data detection in an optical fiber communication network. In their research, the operation data of the optical fiber communication network were first collected, and the data characteristics were extracted. Finally, the optimal value of the entropy target function was calculated by the sample attribute probability, and the optimal value was used to complete the anomalous data detection. Wang et al. [6] proposed data mining technology for Internet industry collaborative innovation platform research. Information technology has therefore been integrated into every corner of production and life. Considering the computing cost, the Internet of things, and intelligent service collaborative innovation as the research object, we studied the combination based on data mining technology of Internet of things and intelligent service collaborative innovation. For the development of intelligent service industry and the improvement of the Internet of things collaborative innovation, we provide valuable theoretical basis. Based on previous research experience, an embedded multifunctional data mining technology based on particle calculation was studied. According to the three characteristics of embedded multifunctional data, data reduction, data standardization, and data balance, the data were preprocessed. The processed data were granulated and analyzed for particle characteristics, including offset, particle density, and intragrain spacer. Furthermore, a particle feature-based neural network was used to classify the data, detect anomalies, and identify faults. Through the data mining of the proposed data mining technology and comparing the methods presented in the literature, the accuracy of the various data mining methods was above 0.9. The innovation point of the studied technology is the data granulation and data particle feature calculation. The embedded multifunctional data wee preprocessed and then the embedded multifunctional data feature extraction was performed. The above extracted features were used as input, and the embedded multifunctional data mining was implemented to achieve data classification, anomaly detection, fault identification, and other goals.

## 2. Embedded Multifunctional Data Mining Technology Based on Particle Computing

### 2.1. General Framework

**Figure 1:**
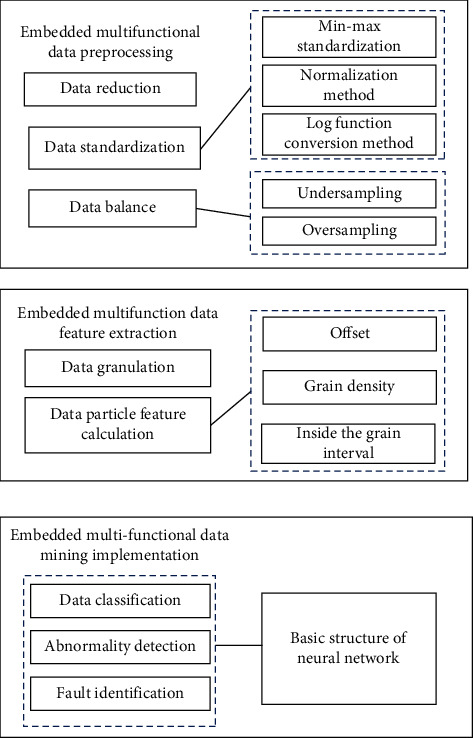
Overall framework of the embedded multifunctional data mining technology.

Embedded multifunctional data are multisourced, heterogeneous, and imbalanced, so mining embedded multifunctional data with widely used data mining technology cannot achieve good results. Facing this situation, it is of great practical significance to study a new data mining technology to deal with embedded multifunctional data. The key to the data mining technology studied here is particle computing, which refers to the division of massive data or information according to certain rules or relationships, thus forming particles. Based on this theory, a data mining technique can be designed for the effective classification of embedded multifunctional data. The general framework of the embedded multifunctional data mining technology is shown in Figure 1.

According to the content shown in Figure 1, embedded multifunctional data mining is a repeated process. If each link fails to achieve the expected results, it must return to the previous step for another adjustment and implementation. Comparing previous models, not all data mining efforts are required to be listed here; for example, data integration can be ignored when there are no multiple data sources in a job. For some multifunctional data, it is a very necessary process to conduct embedded multifunctional data preprocessing, embedded multifunctional data feature extraction based on particle calculation, and embedded multifunctional data mining.

### 2.2. Embedded Multifunctional Data Preprocessing

To realize the effective mining of the embedded multifunctional data, the embedded multifunctional data preprocessing is required first. Preprocessing can effectively reduce the multisourced, heterogeneous, and imbalanced data, improve the data quality, and facilitate mining [7]. Embedded multifunctional data preprocessing includes data reduction, data standardization, and data balancing. Specific analysis was performed for these three preprocessing steps.

#### 2.2.1. Data Reduction

**Figure 2:**
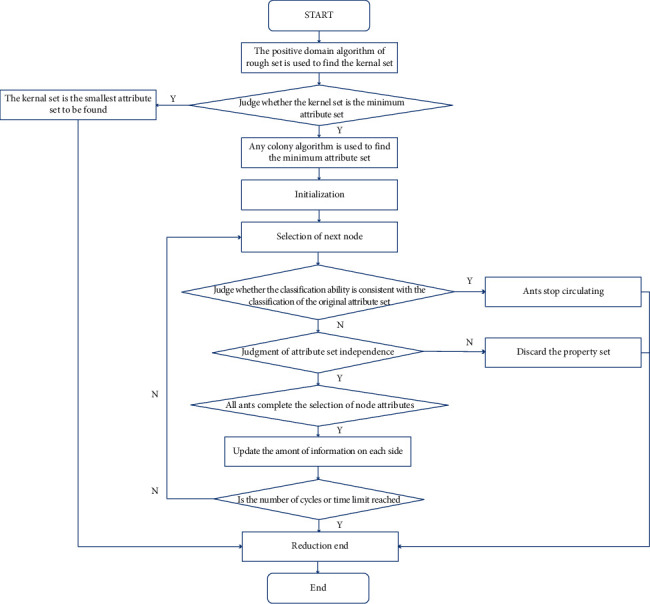
Data reduction process.

Embedded multifunctional data come from multiple different databases, and after pooling the data from multiple databases together, the embedded multifunctional data are formed. The embedded multifunctional data are therefore massive, and such data can be collectively referred to as redundant data [8]. The presence of redundant data will increase the computation and interfere with the data mining results, thus requiring data reduction, as shown in Figure 2.

#### 2.2.2. Data Standardization

Embedded multifunctional data come from multiple different databases, and there is also some heterogeneity, which represents different data dimensions, leading to no synchronous processing between the data [9]. To this end, standardization of embedded multifunctional data is required. The methods for handling this are as follows:

(1) Min-max standardization:
  (1) $$\begin{array}{c} x\prime =\frac{x-\min\left(x\right)}{\max\left(x\right)-\min\left(x\right)}, \end{array}$$
  *x* represents the original embedded multifunctional data, *x*′ represents the standardized embedded multifunctional data, and min(*x*) and max(*x*) represents the minimum and maximum values in the original embedded multifunctional data.
(2) Normalization method:
  (2) $$\begin{array}{c} x\prime =\frac{x-a}{b}, \end{array}$$
  where *a* and *b* represent the mean and standard deviation of the raw embedded multifunctional data.
(3) Log function conversion method:

(3) $$\begin{array}{c} \tilde{x}=\frac{\log_{10}\left(x\right)}{\log_{10}\;\max\left(x\right)}. \end{array}$$

The dimension of embedded multifunctional data is standardized to be unified [10].

#### 2.2.3. Data Balancing

Imbalance is one of the major features of embedded multifunctional data, and the mining of unbalanced data will lead to mining accuracy distortion [11]. For this point, the unbalanced data need to be balanced with the data. Select the undersampling method or oversampling method based on the number of negative and positive samples in the data. The undersampling method is suitable for more negative samples and the oversampling method for more positive samples [12].

*Undersampling*.The undersampling principle refers to the removal of most redundant negative samples to balance with the positive samples [13]. The specific process is as follows:

  Step 1 : enter most class samples, that is, negative samples.
  Step 2 : cluster the negative samples and divide the samples into subsamples of multiple categories.
  Step 3 : calculate the similarity redundancy coefficient between each subsample with the following formula:
  (4) $$\begin{array}{c} S_{K}{=}^{2}\sqrt{d_{i}^{n}\cdot D_{ij}},\;\left(i,j=1,2,\ldots ,n\right). \end{array}$$
*S*_*K*_ represents the similarity redundancy coefficient, *d*_*i*_^*n*^ represents the distance from the subsample *i* to its cluster center, and *D*_*ij*_ represents the Euclidean distance between the subsamples *i*, *j*.
  Step 4 : make the calculated similarity redundancy coefficient into a matrix form.
  Step 5 : delete one of the two subsamples of the minimum similarity redundancy coefficient in the matrix and the corresponding rows and columns in the matrix.
  Step 6 : determine whether the sample deletion requirements are met. If achieved, remove most redundant negative samples and complete the undersampling; otherwise, return to the previous Step 5 until the end requirements are met.

*Oversampling*. The oversampling principle is to select negative samples and then calculate the distance between each Euclidean sample and all the Euclidean distances to determine the *k* nearest neighbors. Finally, the *k* nearest neighbors are selected according to the set sampling fold rate to generate new samples to compensate for the small number of negative samples and to achieve sample balance [14]. The principle formula is as follows:

(5) $$\begin{array}{c} Y_{\text{new}}=y_{k近邻}+\text{rand}\left(0,1\right)\cdot \left(x^{-}-y_{k近邻}\right), \end{array}$$

where *Y*_new_ represents a new sample formed after sampling, *y*_*k*近邻_ represents *k* nearest neighbors, rand(0,1) represents a random number between (0,1), and *x*^−^ represents the original negative sample. The embedded multifunctional data preprocessing is completed to pave the way for the extraction of the embedded multifunctional data features based on particle calculation.

### 2.3. Embedded Multifunctional Data Feature Extraction Based on Particle Calculation

After finishing the embedded multifunctional data, then the embedded multifunctional data feature is extracted. The specific process includes two steps, namely, data granulation and data particle feature calculation [15]. Specific analysis of these two processes is described as follows.

#### 2.3.1. Data Granulation

Data granulation refers to dividing embedded multifunctional data into one data block according to certain rules and relationships. A block of data is called a grain [16]. Through the granulation processing, it is easier to find the rules or characteristics between the data. The data granulation process is described as follows:

Step 1 : enter the embedded multifunctional dataset, noted as *X*={*x*_1_, *x*_2_,…, *x*_*n*_}.
Step 2 : select K data from *X*={*x*_1_, *x*_2_,…, *x*_*n*_}, as the initial category representative, which are recorded as *U*^*h*^={*z*_1_^*h*^, *z*_2_^*h*^,…, *z*_*K*_^*h*^}. Because it is the initial sample, so set *h*=0.
Step 3 : calculate the distance between all samples except the initial category sample and the initial category sample.
Step 4 : according to the proximity principle, divide all the remaining samples into an initial sample category, and get a new cluster, recorded as *P*_*j*_^*h*+1^, *j*=1,2, ..., *K*.
Step 5 : reselect the category representative from step 4 results, noted as *p*_*j*_^*h*+1^.
Step 6 : determine whether *p*_*j*_^*h*+1^ is equal to *U*^*h*^. If equal, end the operation and complete the data granulation; otherwise, set *h*=*h*+1 and return to step 3, and repeat the above steps until the above conditions are fulfilled and the data granulation is completed.
Step 7 : output the granulation results.

#### 2.3.2. Data Particle Feature Calculation

Based on the above divided data particles, the data particle characteristics, including the offset degree, particle density, and interparticle space, are calculated [17]. Calculate these three features.

*Offset.* Offset degree refers to the case of the data particle offset particle center, with the following formula:

(6) $$\begin{array}{c} G_{i,O}=\frac{\sum_{i=1}^{n}f\left[g\left(i,O\right),q\left(O\right)\right]}{n}, \end{array}$$

where *g*(*i*, *O*) represents the accessible distance from the particle *i* to the particle center, *n* represents the particle number, *q*(*O*) represents the particle center capacity, *f* represents the binary mapping function, and *G*_*i*,*O*_ represents the particle *i* offset degree.

*Grain Density*. Particle density refers to the density of the particle distribution. The calculation formula is as follows:

(7) $$\begin{array}{c} \rho =\frac{Bn}{\sum_{i=1}^{n}B\prime }, \end{array}$$

where *ρ* represents the particle density, *B*′ represents the inverse operation representing the average accessible distance between the particle and the particle center, and *B* represents the average accessible distance between the particle and the particle center.

*Inside the Grain Interval*. Inside the grain interval, describe the degree of intimacy between the particles:

(8) $$\begin{array}{c} d=\frac{\sum_{i=1}^{n}\left(B\cdot w/r_{i}\right)}{n}, \end{array}$$

where *r*_*i*_ represents the radius of the particle *i* and *w* represents the degree of membership.

Based on the above process, the embedded multifunctional data feature extraction work based on particle calculation is completed.

### 2.4. Embedded Multifunctional Data Mining Implementation

**Figure 3:**
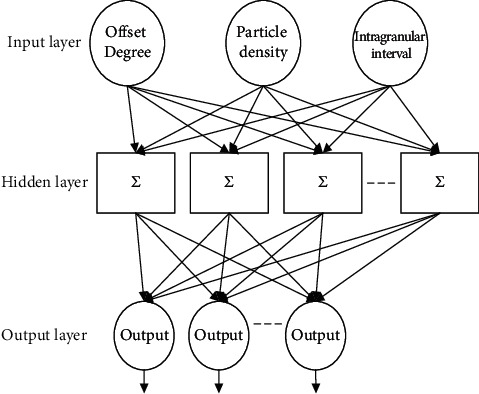
Basic structure of the neural network.

With the above extracted features used as input, the embedded multifunctional data mining is implemented to achieve data classification, abnormality detection, fault identification, and other goals [18]. Here, the neural network method is used to realize the embedded multifunctional data mining. The basic structure of the neural network is shown in Figure 3.

Embedded multifunctional data mining based on the neural network is divided into two steps, namely, training and testing.

1. The training uses the extracted three embedded multifunctional data features, namely, offset, particle density, and the input interval and the output of the processing and operation, and the neural network. If the matching results and the expected set results meet the end conditions, the training will end; otherwise, error backpropagation is performed until the training is successful.
2. The test is based on the former training of the good model, to complete the mining of the test samples.

## 3. Technical Testing and Analysis

For the embedded multifunctional data, the mining technology based on particle computing is taken as an example, which is applied to the network anomaly detection to test the effectiveness of the mining technology. The simulation test platform is Matlab 2016.

### 3.1. Simulation Sample

**Figure 4:**
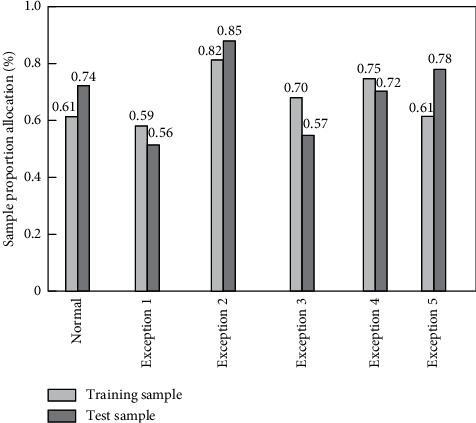
Test sample allocation plot.

Six types of data were selected from the DARPA KDD CUP 99 dataset to form the embedded multifunctional data simulation samples, with a total number of 10,000 samples. The sample proportion allocation is shown in Figure 4.

Since the samples were obtained from the standard DARPA KDD CUP 99 dataset, the preprocessing process was not analyzed in detail.

### 3.2. Embedded Multifunctional Data Particle Feature

**Figure 5:**
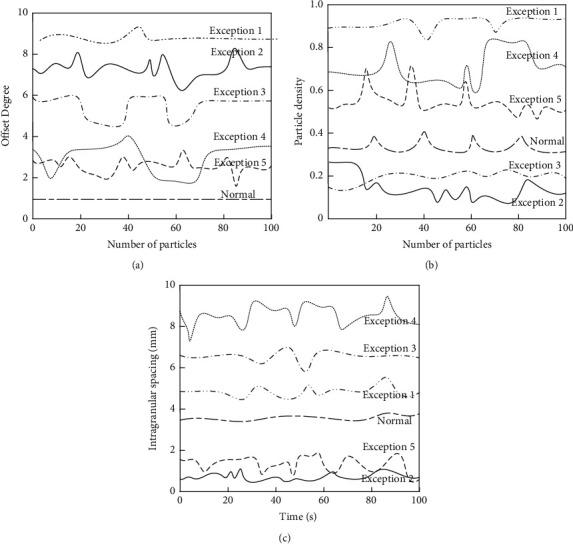
Embedded multifunctional data particle feature. (a) Drift rate. (b) Granule density. (c) Internal particle spacing.

In Section 1, the study was used to granulate the embedded multifunctional data samples and then we calculated the data particle features. The results are shown in Figure 5.

### 3.3. Test Indicators

*G* − *mean* proposed by Kubat is the evaluation index of embedded multifunctional data mining technology. The calculation formula is as follows:

(9) $$\begin{array}{c} G-\text{mean}=\sqrt{\frac{TP}{TP+FN}\times \frac{TN}{TN+FP}}. \end{array}$$

The various index parameters in the equation are derived from the confusion matrix, as shown in Table 1.

*G* − mean takes the value (0,1]; when greater than 0.9, the mining technical accuracy is high.

## 4. Results and Analysis

The neural network was trained using the training samples, and the post-training weights were set to 0.25 and 0.36; the thresholds were set to 1.20 and 1.50. Taking the test sample as input, the trained neural network model was used for embedded multifunctional data mining to obtain anomalous mining results. Finally, the values were calculated from the anomalous mining results, as shown in Table 2*G* − mean.

As can be seen from Table 2, the abnormal mining result of each type of data mining is greater than 0.9, thus indicating that the accuracy of the studied mining technology is high.

In conclusion, the studied mining technology was used to carry out abnormal mining of embedded multifunctional data many times, and in different cases, the results of each type of data mining were all greater than 0.9, showing high accuracy and good results.

## 5. Conclusion

Data mining is the most important issue in big data processing, where categories, rules, and even abnormalities can be found from the data. Current data mining is limited by the embedded multifunctional data features, and the mining accuracy is not high. For the above problems, an embedded multifunctional data mining technique based on particle calculation was studied. This technology has been tested and its effectiveness has been proved. It can cope well with the embedded multifunctional data mining technology based on particle computing, with high accuracy and good modification. However, this study only tested the technology in one field, and therefore, the test results have limitations. Further testing is needed, and in the future, particle calculation in embedded multifunctional data mining can be improved. From the perspective of collaborative innovation, data mining technology innovation ability can also be improved. The key is to face the characteristics of the Internet of Things industry and to explore the technology collaborative innovation process and behavior collaborative interaction mode through in-depth data mining and analysis to develop more intelligent applications.

## Figures and Tables

**Table 1:** Confounding matrix.

Class	Positive class	Negative class
Positive class	TP	FN
Negative class	FP	TN

**Table 2:** *G* − mean values for the statistical results.

Type	1 test	Two tests	Three tests	4 tests	5 tests
Normal	0.925	0.921	0.936	0.945	0..951
Abnormal 1	0.932	0.934	0.935	0.941	0.932
Abnormal 2	0.965	0.952	0.932	0.952	0.934
Abnormal 3	0.951	0.941	0.914	0.953	0.956
Abnormal 4	0.923	0.920	0.922	0.940	0.955
Abnormal 5	0.920	0.923	0.924	0.933	0.942
Average value	0.936	0.932	0.928	0.944	0.936

## Data Availability

The data for all figures used to support the findings of this study are included within the article.
